# A CRISPR Test for Rapidly and Sensitively Detecting Circulating EGFR Mutations

**DOI:** 10.3390/diagnostics10020114

**Published:** 2020-02-19

**Authors:** Jen-Hui Tsou, Qixin Leng, Feng Jiang

**Affiliations:** Department of Pathology, University of Maryland School of Medicine, 10 S. Pine St. Baltimore, MD 21201, USA; jtsou@som.umaryland.edu (J.-H.T.); qleng@som.umaryland.edu (Q.L.)

**Keywords:** cancer, CRISPR, EGFR, mutations, plasma, liquid biopsy

## Abstract

The detection of EGFR mutations in circulating cell-free DNA can enable personalized therapy for cancer. The current techniques for detecting circulating EGFR mutations are expensive and time-consuming with moderate sensitivity. Emerging CRISPR is revolutionizing medical diagnostics and showing a great promise for nucleic acid detection. This study aims to develop CRISPR-Cas12a as a simple test to sensitively detect circulating EGFR mutations in plasma. Serially diluted samples of DNA containing heterozygous EGFR mutations (L858R and T790M) in wild-type genomic DNA are concurrently tested for the mutations by a CRISPR-Cas12a system and droplet digital PCR (ddPCR). The CRISPR-Cas12a system can detect both L858R and T790M with a limit of detection of 0.005% in less than three hours. ddPCR detects the mutations with a limit of detection of 0.05% for more than five hours. Plasma samples of 28 lung cancer patients and 20 cancer-free individuals are tested for the EGFR mutations by CRISPR-Cas12a system and ddPCR. The CRISPR-Cas12a system could detect L858R in plasma of two lung cancer patients whose tissue biopsies are positive for L858R, and one plasma sample of three lung cancer patients whose tissue biopsies are positive for T790M. ddPCR detects L858R in the same two plasm samples, however, does not detect T790M in any of the plasma samples. This proof of principle study demonstrates that the CRISPR-Cas12a system could rapidly and sensitively detect circulating EGFR mutations, and thus, has potential prognostic or therapeutic implications.

## 1. Introduction

Lung cancer has a dismal prognosis with the highest mortality rate among all cancer types. Non–small cell lung cancer (NSCLC) accounts for 85% of all lung cancers. Epidermal growth factor receptor (EGFR) is a transmembrane receptor tyrosine kinase [1]. Activation of EGFR induces the proliferation and growth of cancer cells, and thus, provides a therapeutic target in advanced NSCLC [1]. EGFR-tyrosine kinase inhibitors (EGFR-TKI) can significantly prolong the five-year survival of advanced NSCLC patients harboring EGFR mutations [1]. Osimertinib, a third-generation EGFR-TKI, represents the new standard of care for treatment of EGFR mutated NSCLC patients in 1st line [2]. L858R point mutation of EGFR is a major mutation of the gene, which causes EGFR-TKI sensitivity [1]. T790M point mutation of EGFR is the key source of resistance to first-generation EGFR-TKIs, comprising 70% of acquired resistance. Since the EGFR gene mutations are related to the EGFR-TKI efficacy, patients with advanced lung cancer are recommended to have their tumors tested for the EGFR sensitizing and resistance mutations [1]. However, examining re-biopsy tissue specimens for the mutations is often difficult, because of the invasive procedure that can be harmful to patients with advanced-stage NSCLC. Furthermore, the inability to obtain a sufficient amount of tissue sample and operator variability and lesion heterogeneity are also concerned for molecular analysis of biopsy tissues for the mutations.

Liquid biopsy for detecting plasma circulating tumor DNA (ctDNA) has entered clinical practice in NSCLC for detection of EGFR mutations either at baseline in patients with no available tissue for tumor genotyping or at disease progression after 1st/2nd generation EGFR TKIs [3,4]. Three major techniques, including amplification refractory mutation system-PCR, droplet digital PCR (ddPCR), and next-generation sequencing, are currently available to detect EGFR mutation in plasma as a blood-based minimally-invasive alternative to tissue biopsy. The use of plasma NGS is growing, since it allows the simultaneous evaluation of multiple oncogene drivers (ALK and ROS1 translocations, BRAF mutations, etc.) beyond EGFR mutations and is not inferior to the standard of care tissue genotyping [5]. However, they are labor intensive and time consuming. Furthermore, the techniques require extensive sample manipulation and expensive machinery. Moreover, since ctDNA in plasma is highly fragmented and usually present at a low abundance level, the overall sensitivity of the current platforms is not enough for reliable detection of the circulating EGFR mutations. Therefore, a highly sensitive, rapid, and cost-effective assay is urgently needed to detect the circulating EGFR mutations in clinical settings.

Clustered regularly interspaced short palindromic repeats (CRISPR) are a family of DNA sequences found within the genomes of prokaryotic organisms [6,7,8]. CRISPR-associated (Cas) immune system has been applied in molecular biology to target and cleave specific nucleic acid sequences, and is commonly used in gene editing. Furthermore, upon binding to target double-stranded DNA, Cas proteins can be activated and unleash the nonspecific endoribonuclease activity to degrade the single-stranded DNA, and thus, provides a novel diagnostic approach for nuclei acid detection [6,7,8]. For instance, we have recently demonstrated that a CRISPR-Cas12a-system can sensitively and rapidly detect circulating DNA of human papillomaviruses in plasma without requiring expansive and ancillary machinery [9].

In this study, we investigate whether the CRISPR-Cas12a-system could detect the EGFR sensitizing (L858R) and resistance (T790M) mutations in plasma.

## 2. Materials and Methods

### 2.1. Cell Culture

Cancer cell line (H1975 (ATCC^®^ CRL-5908™) was obtained from the ATCC (Manassas, VA). The cell line was cultured in RPMI-1640 medium with a final concentration of 10% fetal bovine serum according to ATCC’s instruction.

### 2.2. Preparation of EGFR Mutation Reference Standards

H1975 cell line genomic DNA is heterozygous for EGFR T790M mutation and L858R mutation [10]. To prepare mutation reference standards, we serially diluted genomic DNA of H1975 cancer cells in genomic DNA obtained from peripheral blood mononuclear cells of a healthy donor by following mutation allele frequencies of 0.005%, 0.05%, 0.5%, 5% and 50%.

### 2.3. Clinical Specimens

Written informed consent was obtained from all patients under a protocol approved by the Institutional Review Board of the University of Maryland, Baltimore. The project identification code is 27681. The protocol was approved on 25 August 2006. Plasma samples were obtained from 25 patients who were diagnosed with lung adenocarcinoma. EDTA-anticoagulant blood samples were collected at the time of the interview by venipuncture from the consented subjects as previously described [11]. The samples were centrifuged at 750× *g* for 10 min, and the plasma fractions were stored at −80 °C [11]. We also obtained plasma samples of three NSCLC patients from Tissue Solutions (Glasgow, UK). EGFR mutations of the lung cancer patients were determined in their tumor tissues by the Qiagen RGQ PCR with the Rotor-Gene^®^ Q 5plex HRM^®^ instrument (Qiagen) in clinical laboratories. Plasma samples collected from 20 age- and gender-matched cancer-free smokers were used as controls to determine the cut-off level of mutation allele frequency.

### 2.4. Extracted DNA Preparation

DNA was isolated from cultured cells, cultured cells’ supernatants, or human plasma specimens by using the Qiagen DNeasy^®^ Blood and Tissue Kit per each manufacturer’s instructions. Briefly, 200 µL of lysis buffer AL was added to the specimens, and then suspended in 200 µL PBS with 20 µL proteinase K for digestion followed by incubation at 56 °C for 10 min. The mixture was transferred to DNeasy Mini spin column. The flow-through was discarded, and the QIAampMinElute column was transferred to a new 2 mL collection tube, washed with 500 µL of Buffer AW1, and centrifuged at 8000 rpm for 1 min. The same procedure was as AW1 by adding 500 µL of wash buffer (2) AW2 and then centrifuged at 14,000 rpm for 3 min. The spin column was transferred to a new 1.5 mL collection tube. DNA was eluted by adding 30 µL of buffer AE. The column was incubated for one minute at room temperature and then centrifuged at 8000 rpm for one minute.

### 2.5. Polymerase Chain Reaction (PCR)

Five µL DNA was obtained from either the serially diluted samples containing mutated DNA (H1975 cell DNA) or crude human plasma. Each reaction was prepared in a total volume of 50 µL containing 1× TaqMan™ Universal PCR Master Mix, no AmpErase™ UNG (ThermoFisher), 5 µL DNA, and EGFR790 and EGFR858 specific primer pairs (Table 1). PCR was performed using Bio-Rad CFX96 Real-time system (Bio-Rad). Thermal cycle conditions were as follow: An initial incubation at 95 °C for 10 min followed by 40 to 45 cycles of alternating 95 °C for 10 s, 54.7 °C for 10 s and 72 °C for 30 s. A further incubation at 72 °C for 10 min was carried out to complete the extension step.

### 2.6. Fluorescent Readout of CRISPR-Cas12a Activity by Using FAM-Quencher (FQ) Reporters

Five µL of amplified DNA was transferred to a 384-well microplate (Corning). LbCas12a was pre-assembled with an EGFR790 or EGFR858-targeting crRNA at 37 °C for 30 min and diluted in 1X binding buffer (20 mM Tris-HCl, pH 7.5, 100 mM KCl, 5 mM MgCl2, 1mM DTT, 5% glycerol, 50 µg/mL heparin) and custom ssDNA-FQ reporter (IDT). Sequences of guide RNA and reporter substrates were listed in Table 2 [7]. The pre-assembled mixture containing 250nM of LbCas12a, 500nM of crRNA, and 500nM of ssDNA-FQ reporter was added directly to the amplified DNA reactions in total 20 µL volumes. Reactions were proceeded at 37 °C for up to two hours on a fluorescence plate reader (Biotek^®^ Synergy™ H1) with emission filters (λex: 485 nM; λem: 535 nM) for fluorescent readout.

Droplet digital PCR (ddPCR). The ddPCR mixture contained 10 µL of 2× ddPCR™ Supermix for probes, 0.5 µL of 40× TaqMan^®^ dPCR Liquid Biopsy Assays, including pre-formulated of two probes and two primers (Thermo Fisher Scientific) and 5 µL of prepared DNA. A 20 µL reaction was loaded into a 96-well plate (Eppendorf, Hamburg, Germany) and hot-sealed a foil lid by P × 1 PCR plate sealer (Bio-Rad Laboratories). The reaction mixture was applied to the droplet generator with 70 µL of oil and draw through a flow-focusing nozzle to form droplets in 35 µL of oil-in-water mixture. The mixture was transferred to a 96-well droplet plate and heated-sealed using a foil lid. The sealed droplet plate was cycled using a C-1000 touch thermal cycler (Bio-Rad Laboratories) under the following conditions: An initial incubation at 95 °C for 10 min followed by 40 cycles at 94 °C for 30 s, and then at 60 °C for 60 s. A further incubation at 98 °C for 10 min was done to deactivate enzyme activity. After the amplification process, the 96-well PCR plate was read on a QX-200 droplet reader (Bio-Rad Laboratories) and the number of PCR-positive and -negative droplets for each fluorophore (FAM and VIC) were counted by passing them in a single stream through a fluorescence detector. The threshold line was determined by serial dilution of extracted cell DNA from H1975 and was drawn for channel 1 (FAM) and channel 2 (VIC) to separate the two clusters of negative and positive droplets. Wells with droplet events over 9000 were analyzed. The absolute copy numbers in copies/µL units of both mutant and wild-type DNA were analyzed by using QuantaSoft™ Software (Bio-Rad) based on the Poisson distribution.

Statistical analysis. We used Fisher’s exact test and Mann-Whitney U-test to analyze the variables. All statistical tests were two-sided. A *p*-value ≤ 0.05 was statistically significant. We reported the results as two-sided *p*-values with 95% confidence intervals (95% CI). Statistical analysis was performed with Prism (Graphpad Software, San Diego, CA, USA) and Quantasoft (Bio-Rad, Hercules, CA, USA).

## 3. Results

### 3.1. The CRISPR-Cas12a System Has a Higher Sensitivity for Detecting the EGFR Mutations Compared with ddPCR Assay

**Figure 1:**
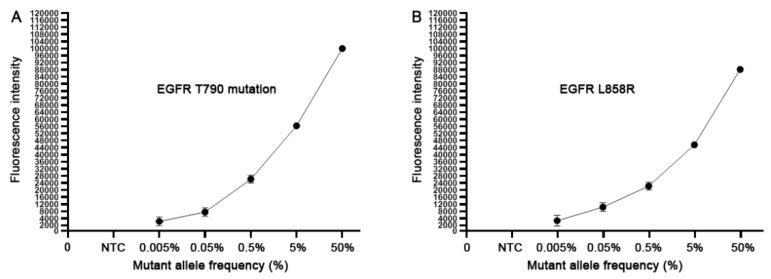
The analytical sensitivity of the CRISPR-Cas12a system. The serially diluted H1975 positive cell line DNA with different mutant allele frequency (%) of 790M (**A**) and L858R (**B**) in human normal genomic DNA was tested. Each concentration of the serially diluted positive cell line DNA was tested in triplicates for three times.

To define the analytic performance of the CRISPR-Cas12a system, we extracted genomic DNA of H1975 cancer cells. H1975 cells have EGRF L858R and T790M heterozygous mutations (50% mutation rate) with 151.5 copies of the mutated EGFR per ng genomic DNA (9,11). Genomic DNA of H1975 cancer cells was serially diluted into human normal genomic DNA with mutant allele frequency ranging from 0.005%, 0.05%, 0.5%, 5% and 50%. Accordingly, the dynamic range of the copy numbers of EGFR T790M DNA and wild-type DNA in the serially diluted samples was from 1:20,000 to 1:20 copies. Each of the serially diluted samples was processed for the CRISPR-Cas12a system and ddPCR at least three times. As shown in Figure 1, the CRISPR-Cas12a system could detect the EGFR mutations over the range of 50% to 0.005%, proposing that the CRISPR-Cas12a system had a detection limit of 0.005% or 1:20,000 copies of mutated DNA in human normal genomic DNA. ddPCR detected the EGFR mutations over the range of 50% to 0.05%, suggesting that ddPCR had a detection limit of 0.05% or 1:2000 copies of mutated DNA in human normal genomic DNA. Thus, the CRISPR-Cas12a system had a higher sensitivity (10 fold) compared to ddPCR for detecting the EGFR mutations in the reference standard DNA samples (*p* < 0.05). Furthermore, the CRISPR-Cas12a system had a significantly smaller mean coefficient of variation (CV) of 0.38–2.35% compared to that (5.49–15.26%) of ddPCR regarding overall and low mutant allele frequencies (0.05%) (All *p* < 0.05). Therefore, the CRISPR-Cas12a system could sensitively and precisely detect the low-abundance EGFR mutations in the specimens.

To determine whether the system could detect circulating EGFR mutations in liquid fluids, we collected cell-cultured supernatants, which mimicked body fluids, of H1975 lung cancer cells. DNA was extracted from the cell supernatants and serially diluted (10-fold) into water. Each diluted sample was tested for the EGFR mutations by using the CRISPR-Cas12a system and ddPCR. For the CRISPR-Cas12a system, 5 µL DNA from each sample was added into the final volume of 50 µL, from which, 5µL reaction was obtained and mixture with LbCas12a, crRNA, and ssDNA FQ reporter. For ddPCR, 5 µL DNA from each sample was added into the final volume of 20 µL, which was processed for the PCR analysis. The CRISPR-Cas12a system had a detection limit of 10 × 10^−3^ ng for L858R and of 10 × 10^−4^ ng for T790M, respectively. ddPCR had a detection limit of 10 × 10^−1^ only for L858R, however, was not able to detect T790M. Therefore, the CRISPR-Cas12a system has a sensitivity that is significantly higher compared with ddPCR for detecting the EGFR mutations in the cell-cultured supernatant specimens.

### 3.2. The CRISPR-Cas12a System Can Rapidly Detect the EGFR Mutations in the Crude Preparation of Cell-Cultured Supernatant

**Figure 2:**
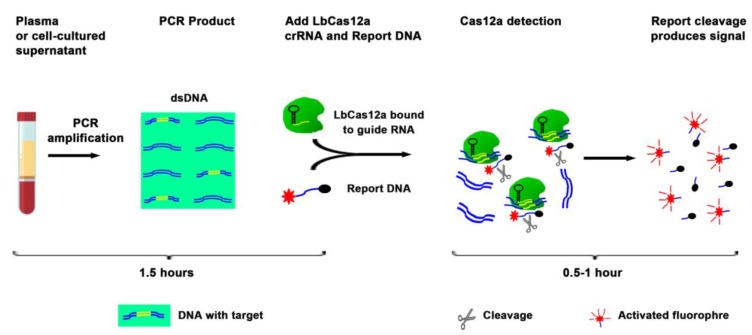
Overview of the CRISPR-Cas12a system to directly target plasma for the detection of EGFR mutations. Plasma or supernatant of cultured cells were processed for simply PCR amplification. The PCR product was a mixture with LbCas12a, crRNA, and ssDNA FQ reporter. The reaction was proceeded for up to two hours at room temperature on a plate reader for fluorescent readout.

The current techniques, including ddPCR, for analysis of DNA mutations, require a large plasma volume to purify a large amount of DNA followed by complex downstream workflow (12). Therefore, the techniques are labor intensive and time consuming. To evaluate whether EGFR mutations could be directly detected in liquid fluids without DNA isolation, we diluted the cell-cultured supernatants in PBS (1:3) containing 0.53% Triton X-100. The mixture was boiled at 95 °C for six minutes, and serially (10-fold) diluted into a vehicle containing Triton X-100 in PBS and processed for simply PCR amplification followed by the CRISPR-Cas12a system (Figure 2, and Table 3). In the crude preparation of 5 µL supernatant, the CRISPR-Cas12a system had a detection limit of 10 × 10^−2^ ng for detection of both EGFR mutations. The process for the CRISPR-Cas12a system for detecting the mutations only took three hours (Table 3). Therefore, the CRISPR-Cas12a system could be used as a rapid test for directly targeting a small volume of liquid material to detect EGFR mutations without requiring complicated procedures.

### 3.3. The CRISPR-Cas12a System Can Sensitively and Rapidly Detect EGFR Mutations in Plasma of Lung Cancer Patients

To determine whether the CRISPR-Cas12a system could detect circulate EGFR mutations in clinical plasma specimens, we obtained plasma samples of 28 lung cancer patients (Table 4). Of the lung cancer patients, four had proven targetable EGFR mutations in their tumor tissues. Of the four lung cancer patients, two had L858R mutation, three had T790 M mutation, and one had both mutations in their tumor tissue specimens (Table 5). Plasma samples of 20 age- and gender-matched cancer-free smokers were used as controls to determine the cut-off for calling mutate positive samples (Table 4).

Both the CRISPR-Cas12a system and ddPCR were successfully performed in the plasma samples of the lung cancer patients and cancer-free individuals. Based on the results generated from plasma samples of the 20 cancer-free individuals, cut-off of mean fluorescence intensity >25,775 and 31,702 was set up for the CRISPR-Cas12a system to determine positive L858R and T790M in plasma, respectively. The cutoff value for ddPCR to determine positive L858R and T790M in plasma was defined as two copies/µL, respectively. According to the cutoff values, the CRISPR-Cas12a system could detect L858R in the two cases whose tissue biopsy specimens were positive for L858R, and T790M in one of the three lung cancer patients whose tissues were positive for T790M (Table 5). ddPCR could detect L858R in the same two plasma samples that were positive by the CRISPR-Cas12a system, however, did not yield a positive result for T790M in any of the three plasma samples from the patients whose tissues were positive for T790M (Table 5). Furthermore, both CRISPR-Cas12a system and ddPCR did not produce positive results in the plasma samples of 24 lung cancer patients whose tumor tissues were negative for the EGFR mutations. Altogether, for detecting circulating L858R, both CRISPR-Cas12a system and ddPCR had 100% sensitivity and 100% specificity, respectively. For detecting circulating T790M, the CRISPR-Cas12a system had 33.33% sensitivity and 100% specificity. In addition, the CRISPR-Cas12a system only needed 20 µL plasma and took approximately three hours, whereas, ddPCR required at least 200 µL plasma and had a turnaround time of more than five hours. Therefore, the CRISPR-Cas12a system might have a higher sensitivity for rapidly detecting circulating T790M in the plasma samples of lung cancer patients.

## 4. Discussion

The detection of EGFR mutations in ctDNA could guide treatment decision in lung cancers. However, the current techniques are time-consuming, labor-intensive, and expensive. Furthermore, the mutated DNA is short, fragmented, and present at a very low concentration in the extremely high background of non-mutant DNA. The techniques that can detect EGFR mutations with high sensitivity are needed. CRISPR-Cas biology has revolutionized the field of molecular diagnostics for various diseases, since it has an extremely high sensitivity for detection of nucleic acids [12]. In this proof of principle study, we developed the CRISPR-Cas12a system as a novel assay that could sensitively detect EGFR mutation in ctDNA of plasma specimens. The detection limit of the CRISPR-Cas12a system was significantly higher than ddPCR in serially diluted samples DNA containing EGFR mutations. Consistently, the CRISPR-Cas12a system had a higher sensitivity for detecting T790M in plasma of lung cancer patients compared with ddPCR. Furthermore, CRISPR-Cas12a could directly target plasma for detecting circulating EGFR mutations without extra steps, such as DNA extraction. The turnaround time of the CRISPR-Cas12a system was significantly shorter than that of ddPCR, implying that it could be used as a rapid test for EGFR mutation detection. In addition, the CRISPR-Cas12a system only required a very small amount of plasma (20 µL); however, produced a higher sensitivity, compared with ddPCR, which needed 200 µL or more plasma for DNA isolation. Without requiring extensive sample manipulation and expensive machinery, the CRISPR-Cas12a system might have the potential to be developed as an accurate and portable diagnostic test for diagnosis and prognosis of lung cancer. Moreover, only needing a simple and easily accessible microplate reader, the CRISPR-Cas12a system could provide a fluorescence-based assay for quantitative measurement of EGFR mutations. Therefore, the CRISPR-Cas12a system would overcome the obstacles of the previous platforms and provides a highly sensitive, convenient and cost-effective assay for the detection of plasma EGFR mutations in clinical practice.

The study has some limitations. First, although having 100% for the detection of L858R in plasma, the CRISPR-Cas12a system has only 33% sensitivity for the detection of T790M, which is lower than those (50–65%) that were previously reported [13,14]. The different sensitives may be due to that previous studies analyzed more than one plasma sampling from individual patients after treatment, and at least one of the serial plasma samples showed positive detection of EGFR mutation. However, the present study only tests one plasma sampling from individual lung cancer patients. Therefore, sufficiently large sample size is needed to prospectively validate the clinical significance of the CRISPR-Cas12a system for the detection of circulating EGFR mutations. Second, current ASCO/CAP/IASLC guidelines recommend molecular testing for EGFR mutations, ALK and ROS1 rearrangements, but also for BRAF mutations and exon 14 mutations, HER2 mutations, and RET rearrangements [15]. NGS analysis of tissue and plasma specimen has been shown to be more cost effective than single gene testing in NSCLC [16,17]. For example, a plasma NGS test (Guardant360) could detect guideline-recommended biomarker-positive patients at a rate similar to physician discretion standard-of-care tissue genomic testing [18]. It has high tissue concordance and significantly faster return of results, and thus, could lead to more complete genotyping of the guideline-recommended biomarkers in more patients [18]. In addition, with the upfront use of osimertinib that is associated with different mechanisms of resistance, detection of T790M mutation becomes less important [19,20,21]. However, the CRISPR-Cas12a system only detects EGFR L858R and T790M. Further developing the CRISPR-Cas12a system as a plasma test allowing simultaneous detection of the multiple mutations is needed.

## 5. Conclusions

In sum, this proof of principle study demonstrates that the CRISPR-Cas12a system could rapidly and sensitively detect circulating EGFR mutations. Although having some limitations, this method might be used for rapid EGFR mutations evaluation and has some utility in previous studies [22]. Nevertheless, a comprehensive validation of the novel assay in large sample studies is required.

## Figures and Tables

**Table 1:** Sequences for PCR primers.

Primer’s Name	Sequences (5′–3′)
EGFR790_F	TCACCTCCACCGTGCATTTCATCA
EGFR790_R	TTGCGATCTGCACACACCAGTTGA
EGFR858_F	GTCAAGATCACAGATTTTGGGC
EGFR858_R	GCCTCCTTCTGCATGGTATT

**Table 2:** Sequences of guide RNA and reporter substrates.

Guide RNA’s Name	Sequence (5′->3′)
LbCas12a crRNA-EGFR790-C	UAAUUUCUACUAAGUGUAGAUAUCACGCAGCUCAUGCC
LbCas12a crRNA-EGFR790-T	UAAUUUCUACUAAGUGUAGAUAUCAUGCAGCUCAUGCC
LbCas12a crRNA-EGFR858-T	UAAUUUCUACUAAGUGUAGAUGGCUGGCCAAACUGCUG
LbCas12a crRNA-EGFR858-G	UAAUUUCUACUAAGUGUAGAUGGCGGGCCAAACUGCUG
Substrate’s name	Sequence (5′->3′)
ssDNA-FQ reporter	/56-FAM/TTATT/3IABKFQ/

**Table 3:** Comparison of detecting *EGFR* mutations with CRISPR-Cas12a and ddPCR.

Detecting Method	CRISPR-Cas12a			ddPCR		
**DNA Preparation**	20 µL of liquid materials were diluted 1:3 in PBS containing 0.53% Triton X-100 to the final volume of 60 µL, and boiled to 95 °C for 6 min.			200 µL of liquid materials were extracted by using the Qiagen DNeasy^®^ Blood and Tissue kit per each manufacturer’s instruction, and the final volume was 20 µL. Total processing time was 30 min.		
**Amplification Signal**	5 µLDNA was added into the final volume of 50 µL for PCR and total processing time was 1 h and 30 min.			5 µLDNA was added into the final volume of 20 µL for droplet formation and PCR. Total processing time was 2 h.		
**PCR Condition**	Volume of DNA		5.0	Volume of DNA		5.0
	Nuclease-free water		15.2	Nuclease-free water		4.5
	10 M EGFR F primer		2.4	40× Taqman Liquid Biopsy dPCRAssay (Probe and primers)		0.5
	10 M EGFR R primer		2.4	2× ddPCR™ Supermix for Probes, No AmpErase UNG/ dUTP		10
	TaqMan 2× Universal PCR Master Mix, no AmpErase UNG		25	Total volume per reaction		20
	Total volume per reaction		50			
	95.0 °C	10 min	1 cycle	95.0 °C	10 min	1 cycle
	95.0 °C	10 sec	40–45 cycles	94.0 °C	30 sec	40 cycles
	54.7 °C	10 sec		60.0 °C	1 min	
	72.0 °C	30 sec		98.0 °C	10 min	1 cycle
	72.0 °C	10 min	1 cycle	4.0 °C		1 cycle
	4.0 °C		1 cycle			
**Volume of Detection**	5 µL from a 50 of µL PCR mixture was added directly to the pre-assembled mixture in total 20 µL volumes for fluorescence detection.			Whole 20 µL of ddPCR mixture was applied for copy numbers detection		
**Pre-Assembled Mixture**	250 nM of LbCas12a, 500 nM of targeting crRNA and 500 nM of ssDNA-FQ reporter.			No.		
**Specificity From**	1. Primer pairs. 2. LbCas12a and targeting crRNA.			1. Primer pairs. 2. Taqman probe.		
	Specific amplification of the product with specific CRISPR-Cas12a target binding.			Specific amplification of the product with labeling a target-specific hydrolysis probe.		
	Specific CRISPR-Cas12a target binding activates collateral activity to unleash indiscriminate single-stranded DNA (ssDNA) cleavage activity and further amplify the detection signal.			If the target sequence is present, the probe anneals and is cleaved as the primer is extended. This cleavage of the probe separates the reporter dye from quencher dye, increasing the reporter dye signal.		
**Time for Detection**	From 30 min up to 1 h.			At least 1h and 30 min.		

**Table 4:** Demographics and diagnostic characteristics of 28 lung cancer patients and 20 cancer-free individuals.

	28 Lung Cancer Patients *	20 Cancer-Free Individuals
Median age, years (min, max)	65 (37, 79)	66 (36, 79)
Sex, *n* (%)		
Male	7 (25%)	5 (25%)
Female	21 (75%)	15 (75%)
Race, *n* (%)		
White	20 (71.4%)	14 (70.0%)
Black	3 (10.7%)	2 (10.0%)
Asia	5 (17.9%)	4 (20.0%)
Smoking status		
Yes	12	8
No	13	9
Unknown	3	2
Histology	Adenocarcinoma.	
TNM Stage I		
III	5 (17.9)	
IV	23 (82.1)	

* Of the lung cancer patients, four had proven targetable EGFR mutations in their tumor tissues.

**Table 5:** Mutation status of the four lung cancer whose tissue biopsies were positive for T790M or L878R.

Patient	Mutations Detected in Tissues	Mutations Detected in Plasma by the CRISPR	Mutations Detected in Plasma by ddPCR
1	T790M	T790M (Mean fluorescence intensity: 100000)	
2	T790M		
3	L858R	L858R (Mean fluorescence intensity: 99493)	L858R (10.13/µL)
4	T790M, L858R	L858R (Mean fluorescence intensity: 31419)	L858R (2 copes/µL)

The cut-off of mean fluorescence intensity >25,775 and 31,702 was set up in 20 age and gender-matched cancer-free smokers for the CRISPR-Cas12a system to determine positive L858R and T790M in plasma, respectively. The cutoff value for ddPCR to determine positive L858R and T790M in plasma was defined as two copies/µL, respectively.
